# A direct comparison of divalent metal-ion transporter (DMT1) and hinokitiol, a potential small molecule replacement

**DOI:** 10.1007/s10534-019-00207-2

**Published:** 2019-07-31

**Authors:** Michael D. Garrick, Laura M. Garrick, Lin Zhao, James F. Collins, Joleen Soukup, Andrew J. Ghio

**Affiliations:** 1 Department of Biochemistry, University at Buffalo, Buffalo, NY USA; 2 Department of Pediatrics, University at Buffalo, Buffalo, NY USA; 3 Food Science & Human Nutrition Department, University of Florida, Gainesville, FL USA; 4 National Health and Environmental Effects Research Laboratory, Environmental Protection Agency, Chapel Hill, NC USA

**Keywords:** Iron homeostasis, Ferrous, Ferric, Gene therapy, Chelator

## Abstract

Hinokitiol, a natural lipophilic chelator, appears capable of replacing several iron transporters after they have been genetically ablated. Divalent metal-ion transporter (DMT1) is the major iron importer in enterocytes and erythroblasts. We have compared DMT1 and hinokitiol in multiple fashions to learn if the smaller molecule is a suitable substitute using two HEK293 cell lines engineered to overexpress different isoforms of DMT1. Both the macromolecule and the lipophilic chelator enable import of ferrous ions into HEK293 cells. Hinokitiol also mediates ferric ion import but DMT1 cannot do so. While DMT1 can also import Mn^2+^ ions, hinokitiol lacks this ability. The Michaelis–Menten analysis for kinetics of macromolecular catalysis is also suitable for hinokitiol-supported iron import. To compare hinokitiol to DMT1 relative to other metal ions that DMT1 can transport, we employed an organic extraction procedure with which we initially matched the results obtained for Fe^2+^, Fe^3+^ and Mn^2+^, and then showed that multiple other cations were unlikely to enter via hinokitiol. The small chelator thus shares some functional properties with DMT1, but distinct difference were also noted.

**Electronic supplementary material:**

The online version of this article (10.1007/s10534-019-00207-2) contains supplementary material, which is available to authorized users.

A recent, ingenious proposal (Grillo et al. 2017) argues that small molecule replacement is a potential therapeutic modality when genetic defects eliminate catalytic or transport activity of proteins to obstruct a step in a metabolic pathway. This proposal opens an alternative to gene therapy with the authors suggesting that a natural product that acts as an iron chelator, hinokitiol, can potentially substitute for several iron transporters.

The specific rationale (Grillo et al. 2017) for the Burke group’s use of hinokitiol was “Deficiencies of passive ion-transport proteins cause many human diseases … “(yet) “the corresponding active ion-transport proteins typically remain functional,” (so) “there may be a buildup of ion gradients upstream of the membranes that normally host these missing proteins.” They argued that a smaller molecule like hinokitiol could serve as a channel to relieve this buildup. Their paper supported this rationale by showing that hinokitiol could substitute for divalent metal-ion transporter (DMT1) and other iron transport proteins when they were deficient.

Over-expression of DMT1 in HEK293 cells (Garrick et al. 2006a) could be recast in similar terms because we had originally selected HEK293 cells for having very low iron-transport activity thus implying modest endogenous DMT1 expression. We then stably transfected the cells to create 2 cell lines representing all 4 of the N- and C-termini of DMT1 isoforms and subject to doxycycline inducible over-expression. Two major DMT1 mRNA isoforms vary by starting in exons 1A or 1B; the protein encoded by the former has its N-terminus extended to include exon 1A while the latter does not get translated so its N-terminus starts in exon 2. Two other isoform variants depend on whether poly-A attaches after exon 16 or 17; the 3′ UTR of exon 16 includes an iron responsive element (IRE) but the IRE is absent when exon 17 is present. Although IREs are stem loop structures in mRNA, the respective distinct protein C-termini receive designations: +IRE or –IRE, respectively. Having cells that would overexpress 1A/+ IRE DMT1 or 1B/− IRE DMT1 in a regulated fashion allowed us to characterize the DMT1 isoforms functionally and kinetically. In addition, this approach enabled us to learn more about other metal ions that DMT1 transports (Arredondo et al. 2014; Davidson et al. 2005; Garrick et al. 2003, 2006a, b; Jiang et al. 2013; Roth and Garrick 2003), largely confirming earlier suspicions based on induced currents (Gunshin et al. 1997). This promiscuity was also established by direct measurements subsequently (Illing et al. 2012). Some metal ions like Cu^1+^ remain controversial. These results also lead one to wonder about whether kinetic analyses leading to a Michaelis constant (Km) could be applied to hinokitiol and what other metal ions could rely on hinokitiol to gain entry.

If one advocates using hinokitiol to replace defective DMT1, then a direct comparison of functioning DMT1 and hinokitiol needs to be made. We set out to do so for the most physiologically relevant metal ion, Fe^2+^ as well as Fe^3+^ (the main dietary form of nonheme iron). This study asks how well hinokitiol recapitulates iron transporter(s) (here DMT1) for which it could be substituted. So issues are for which metal ion gradients can the smaller molecule allow entry into cells and how well can the Km concept apply to it.

## Results

### Import assay comparisons

**Fig. 1:**
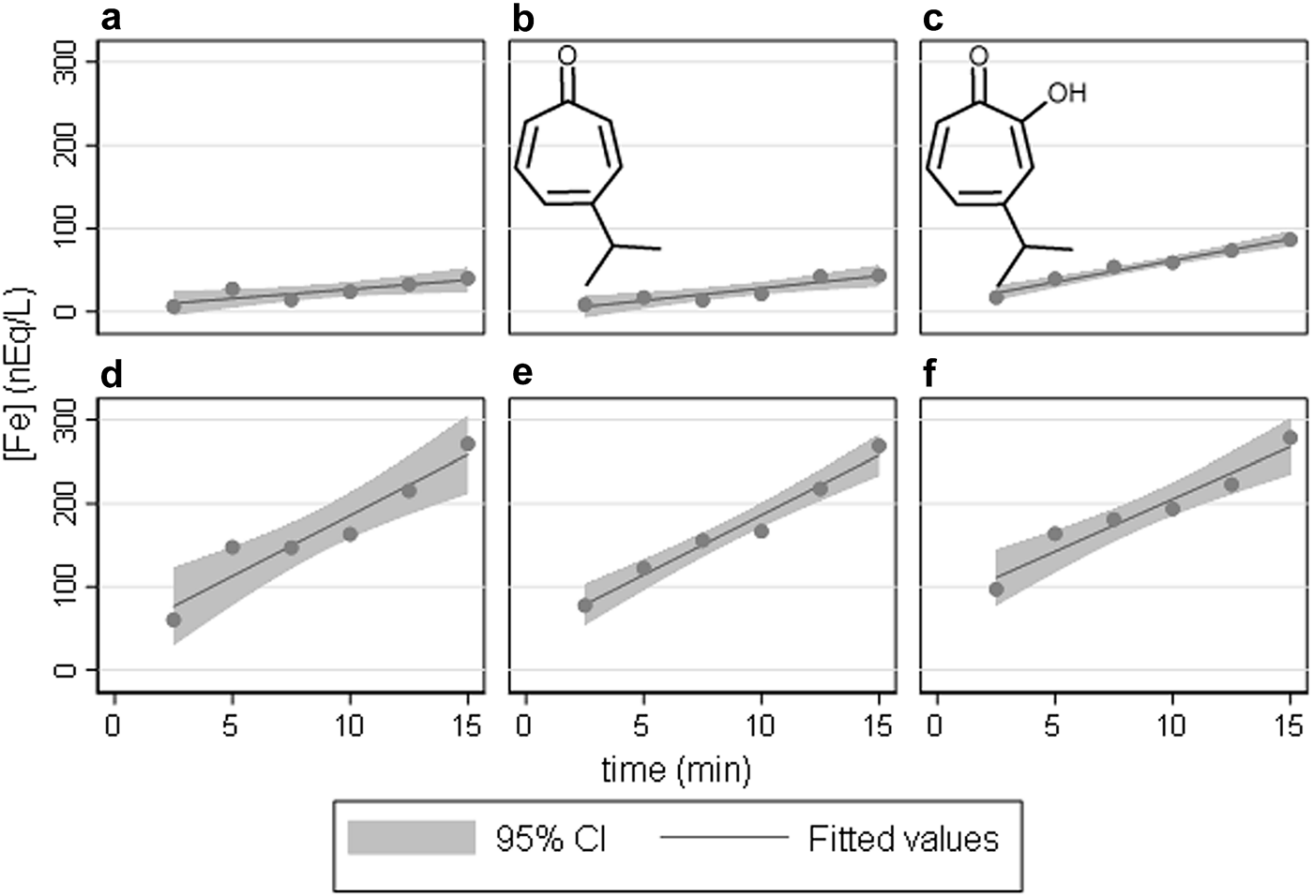
Hinokitiol allows ^59^Fe^2+^ entry into HEK293 cells capable of expressing rat 1A/+ IRE DMT1. Linear regression generated a fit and 95% confidence interval (CI) for each panel. **a** Entry reliant on endogenous transport. **b** Entry reliant on C2d represented structurally by the inset. **c** Entry reliant on hinokitiol represented structurally by the inset. **d** Entry reliant on induced DMT1 expression. **e** Entry reliant on induced DMT1 expression plus C2d. **f** Entry reliant on induced DMT1 expression plus hinokitiol. Comparison of panels **a** and **d** reveals a × 6.5 increase in rate (*P* = 0.0001 by 1XANOVA) due to entry of Fe^2+^ dependent on increased expression of DMT1. Similarly, panels **b** and **e** compare entry with C2d to entry with increased DMT1 plus C2d; DMT1 provides a × 4.8 increase in rate (*P* < 0.00005 by 1XANOVA). Comparison of panels **a** and **b** reveals no significant difference (*P* = 0.94 by 1XANOVA); while comparison of panels **d** and **e** also reveals no significant difference (*P* = 0.95 by 1XANOVA). Both comparisons confirm that C2d does not support entry of Fe^2+^ and is thus an appropriate control. The key comparisons are of the panel **c** (hinokitiol) to its controls—**a** (endogenous) or **b** (C2d); they reveal that hinokitiol stimulated entry by × 2.3 and × 1.8, respectively (*P* = 0.0003 and *P* = 0.0001 by 1XANOVA). (Other comparisons and corrections for multiple comparison are omitted because the focus is only on the most experimentally relevant comparisons.)

Figure 1 compares uptake of ferrous ions by HEK293 cells for a series of situations that illustrate how DMT1 can release an external/internal concentration gradient to generate cellular uptake and that hinokitiol is effective in the same conditions whereas C2deoxyhinokitiol (C2d, an inactive control for hinokitiol) is ineffective. This effectiveness demonstrates that hinokitiol can replace DMT1 for import of ferrous iron.

**Fig. 2:**
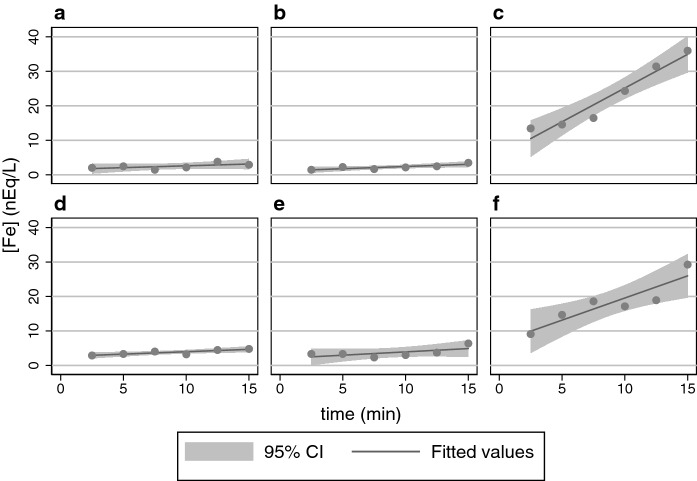
Hinokitiol allows ^59^Fe^3+^ entry into HEK293 cells capable of expressing mouse 1B/− IRE DMT1. Linear regression generated a fit and 95% confidence interval (CI) for each panel. **a** Entry reliant on endogenous transport. **b** Entry reliant on C2d. **c** Entry reliant on hinokitiol. **d** Entry reliant on induced DMT1 expression. **e** Entry reliant on induced DMT1 expression plus C2d. **f** Entry reliant on induced DMT1 expression plus hinokitiol. Comparison of panels **a** and **d** reveals a × 1.3 increase in rate (*P* = 0.0021 by 1XANOVA) due to entry of Fe^3+^ dependent on increased expression of DMT1. This modest but significant increase can likely be attributed to the presence of a surface ferrireductase and very tight experimental control of the assay rather than Fe^3+^ imported by DMT1. Similarly, panels **b** and **e** compare entry with C2d to entry with increased DMT1 plus C2d; DMT1 provides a × 1.5 increase in rate (*P* = 0.001 by 1XANOVA). Again the difference is likely to be attributable to the presence of a surface ferrireductase and very tight experimental control of the assay. Comparison of panels **a** and **b** reveals no significant difference (*P* = 0.51 by 1XANOVA); while comparison of panels **d** and **e** also reveals no significant difference (*P* = 0.83 by 1XANOVA). Both comparisons confirm that C2d does not support entry of Fe^3+^ and is thus an appropriate control. The key comparisons are of the panel **c** (hinokitiol) to its controls—**a** (endogenous) or **b** (C2d); they reveal that hinokitiol stimulated entry by × 18.6 and × 15.2, respectively (*P* = 0.0001 and *P* = 0.0001 by 1XANOVA). (Other comparisons and corrections for multiple comparison are omitted because the focus again is only on experimentally relevant comparisons.)

Results similar to Fig. 1 were obtained with HEK293 cells capable of expressing mouse 1B/− IRE DMT1 (not shown). Our results with hinokitiol confirm and extend those obtained by Grillo et al. (2017), supporting hinokitiol as a potential treatment modality for DMT1 deficiency and the concentration gradient rationale offered. DMT1 fails to transport Fe^3+^ unless supported by a ferrireductase to convert it to Fe^2+^ (Conrad et al. 2000; Fleming et al. 1998; Mackenzie and Garrick 2005), whereas hinokitiol enables Fe^3+^ entry (Grillo et al. 2017). We tested Fe^3+^ entry in this HEK293 system (Fig. 2).

**Fig. 3:**
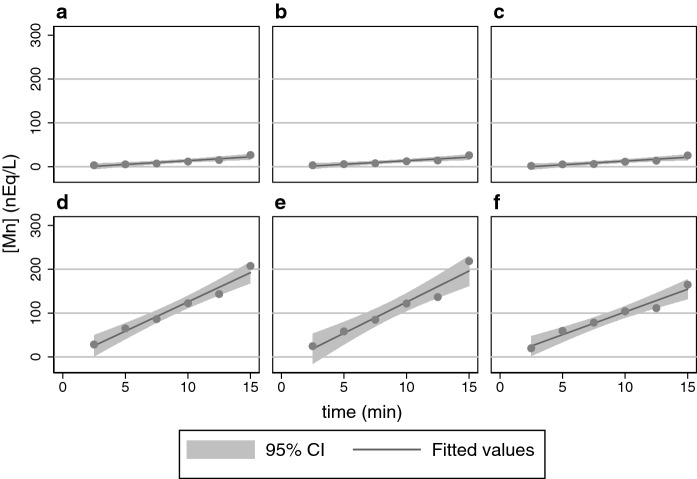
Hinokitiol does not facilitate ^54^Mn^2+^ entry into HEK293 cells capable of expressing mouse 1B/− IRE DMT1. Linear regression generated a fit and 95% CI for each panel. **a** Entry reliant on endogenous transport. **b** Entry reliant on C2d. **c** Entry reliant on hinokitiol. **d** Entry reliant on induced DMT1 expression. **e** Entry reliant on induced DMT1 expression plus C2d. **f** Entry reliant on induced DMT1 expression plus hinokitiol. Comparison of panels **a** and **d** reveals that induced DMT1 provides a substantial × 7.7 increase in rate over endogenous entry of Mn^2+^ (*P* = 0.0003 by 1XANOVA). Similarly, panels **b** and **e** compare entry with C2d to entry with increased DMT1 plus C2d; DMT1 provides an × 8.8 increase in rate (*P* = 0.0007 by 1XANOVA). Comparison of panels **a** and **b** reveals no significant difference (*P* = 0.99 by 1XANOVA); similarly comparison of panels **d** and **e** reveals no significant difference (*P* = 0.86 by 1XANOVA). The key comparisons are of panel **c** (hinokitiol) with its controls—panel **a** (endogenous) or **b** (C2d); they reveal that hinokitiol did not affect entry (*P* = 0.65 and *P* = 0.65, respectively by 1XANOVA). Interestingly, hinokitiol marginally depressed Mn uptake when DMT1 was over-expressed with uptake in the lower right panel 78% of that in the lower left panel (*P* = 0.03 by 1XANOVA) or 71% when compared to that in the lower middle (*P* = 0.10 by 1XANOVA). We did not pursue this effect, given its marginality. (Other comparisons and corrections for multiple comparison are omitted because the focus is only on experimentally relevant comparisons.)

The ordinate for Fig. 1 is × 7.5 greater than that for Fig. 2 to allow coverage of the increased expression of DMT1 in the former; so one should recognize that hinokitiol actually imports Fe^2+^ faster than Fe^3+^. Similar results were obtained with HEK293 cells capable of expressing rat 1A/+ IRE DMT1. The results with hinokitiol confirm and extend those obtained by Grillo and colleagues (Grillo et al. 2017) to indicate that hinokitiol can enable the import of iron in its ferric form whereas DMT1 does not do so (Conrad et al. 2000). Nevertheless, the import is faster with ferrous ions as they had also noted after incorporating hinokitiol into liposomes (Grillo et al. 2017). Because DMT1 supports uptake of Mn^2+^, we then also tested whether hinokitiol did so as well (Fig. 3).

Results similar to Fig. 3 were obtained with HEK293 cells capable of expressing rat 1A/+ IRE DMT1. The results with hinokitiol confirm and extend those obtained before (Grillo et al. 2017) to indicate that hinokitiol does not enable Mn^2+^ import. Although it is clear that hinokitiol can rely on concentration gradients of external/internal Fe^2+^, its behavior is clearly distinct in this role from that of DMT1 because DMT1 can similarly import Mn^2+^ based on concentration gradients of external/internal Mn^2+^, gradients that are not utilized by hinokitiol. Moreover, hinokitiol directly permits Fe^3+^ import while DMT1 does not.

### Kinetics for transport by hinokitiol

Transporters like DMT1 act as macromolecular catalysts similar to enzymes. As such they follow Michaelis–Menten kinetics (Eq. 1) (Michaelis and Menten 1913).

1 $$ {\text{E}} + {\text{S}} \rightleftharpoons {\text{ES}} \to {\text{E}} + {\text{P}} $$

where E = enzyme (or transporter for this analysis); S = substrate (here, extracellular Fe); ES = enzyme/substrate complex; P = product (here, intracellular Fe) and rate constants k_f_, k_r_ and k_cat_ are assigned to the forward and reverse reactions plus to conversion to P, respectively.

Equation 1 is usually analyzed further

2 $$ {\text{v}} = d[{\text{P}}]/d{\text{t}} = {{\text{Vmax} \cdot \left( {\left[ {\text{S}} \right]} \right)} \mathord{\left/ {\vphantom {{\text{Vmax} \cdot \left( {\left[ {\text{S}} \right]} \right)} {\left( {{\text{Km}} + \left[ {\text{S}} \right]} \right)}}} \right. \kern-0pt} {\left( {{\text{Km}} + \left[ {\text{S}} \right]} \right)}} $$

where v = the initial velocity for formation of product; Vmax = the maximal velocity and Km = (k_r_ + k_cat_)/k_f_. This result assumes that [S] > [E] so that one sees a saturable process but the left hand side of Eq. 1 is symmetrical in E and S.

**Fig. 4:**
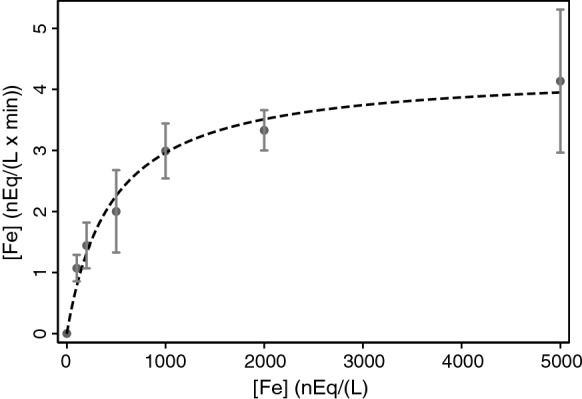
Fe^2+^ entry as a function of initial external [Fe^2+^] in the presence of 1000 nM hinokitiol for HEK293 cells capable of expressing mouse 1B/− IRE DMT1. The points plotted represent the rates ± 95% CI (error bars) calculated by linear regression from plots like those in Fig. 1 except that only 3 time points were used. Nonlinear least squares regression fitting to a Km curve generated the fitted values for the curve (—) with the estimates for Km = 455 ± 86 nEq/L and Vmax = 4.3 ± 0.2 nEq/(L × min). The Stata command used was nl (rate = ({Vm = 5} × Input_Fe2)/({Km = 400} + Input_Fe2)) where rate was the y variable and InputFe2, the x variable for the figure. The assigned initial value estimates (5, 400) affect how rapidly the nl (nonlinear least squares) command converges but not the final values on which it converges

This property of transporters encouraged us to compare hinokitiol head-to-head to DMT1 in such an assay of transport or catalysis as one can argue that the concentration response to Fe^2+^ for a channel like hinokitiol could also follow Michaelis–Menten kinetics. We therefore examined this response for hinokitiol with time dependency graphs like those in Fig. 1 as the basis for the initial velocity in each point (Fig. 4). Prior to being able to use nonlinear least squares fits to estimate Km and Vmax, various transformations were used to linearize the graphs of which the classical approach (Lineweaver and Burk 1934) takes the reciprocals of both sides of Eq. 2 to give a linear result:

3 $$ 1/{\text{v}} = \left( { 1/{\text{Vmax}}} \right) + \left( {{\text{Km}}/{\text{Vmax}}} \right) \cdot \left( { 1/\left[ {\text{S}} \right]} \right) $$

Supplementary Fig. S1 shows a Lineweaver–Burk plot for the same data with an acceptable linear fit. The differences in the estimates for Km (242 vs 455) and Vmax (3.5 vs 4.3) are mostly attributable to the inverse weighting in a reciprocal plot so we chose to use the latter two values in Table 1. The fit to Michaelis–Menten kinetics (Fig. 4) for 1000 nM hinokitiol as a catalyst is suitable even though the [S] values are in a range comparable to those for [E] so we used the same approach to ascertain the Km and the Vmax when 500 nM hinokitiol was present and [Fe^2+^] was varied (Table 1) and obtained an essentially identical Km while the Vmax was essentially halved. We also applied this approach to analyses for Fe^3+^ entry (Table 1) and observed that hinokitiol had a decreased apparent affinity for it but allowed more rapid entry. In contrast, the Burke group found by more direct measurements that the affinity for Fe(III) was much higher than for Fe(II) (Grillo et al. 2017). Noting that the left side of Eq. 1 is symmetrical in E and S, we tested if hinokitiol (E) could also be evaluated by this approach (Table 1) and obtained kinetic parameters again.

**Table 1:** Michaelis–Menten analyses for hinokitiol and Fe

Concentration constant	Concentration varied	Vmax	Km
500 nM hinokitiol	Fe^2+^	2.2 ± 0.1	448 ± 66
1000 nM hinokitiol	Fe^2+^	4.3 ± 0.2	455 ± 86
500 nM hinokitiol	Fe^3+^	24.1 ± 3.3	1790 ± 500
1000 nM hinokitiol	Fe^3+^	87 ± 8	3010 ± 510
200 nM Fe^2+^	Hinokitiol	5.8 ± 1.2	6000 ± 2400
500 nM Fe^2+^	Hinokitiol	15.7 ± 5.4	13,200 ± 7000
200 nM Fe^3+^	Hinokitiol	6.2 ± 1.1	1620 ± 848
500 nM Fe^3+^	Hinokitiol	10.1 ± 0.6	545 ± 117

Data for 1000 nM hinokitiol resulted from fitting the data in Fig. 4 as described in its legend. Remaining rows came from similar analyses (not shown) with the concentration of hinokitiol unvaried in each of the top four rows while that for iron was unvaried in each of the last four rows. Vmax units are nEq/L min; km units are nEq/L

The results with hinokitiol extend those obtained by Grillo et al. (2017), demonstrating that this small molecule can substitute for DMT1 for Fe^2+^ entry into cells provided that there is a concentration gradient of the substrate across the plasma membrane. They also show that saturable kinetics apply to using hinokitiol in place of DMT1 with Fe^2+^ entry into cells. Hinokitiol can also do this for Fe^3+^ entry unaided whereas DMT1 requires reduction of the substrate prior to entry and again a Km analysis is applicable. Remarkably, one can also hold the initial concentration of ferrous or ferric ions constant, vary the hinokitiol concentration and fit the results to Michaelis–Menten kinetics. The saturable behavior could be due to the symmetry of E and S on the left hand side of Eq. 1 or to the solubility of hinokitiol in the relevant membrane (here the plasma membrane).

### Assessing hinokitiol’s chelation of metal ions

**Fig. 5:**
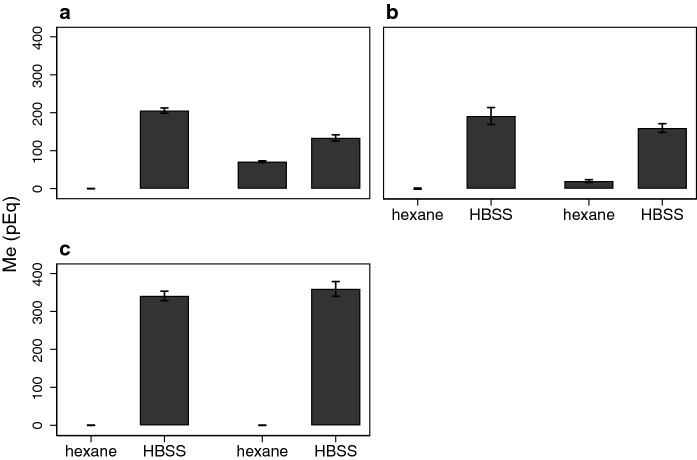
Hinokitiol supports extraction of ^59^Fe^2+^and ^59^Fe^3+^ but not ^54^Mn^2+^. **a** Fe^2+^. The bars’ lengths indicate how much of the original 200 nEq/L of ^59^Fe^2+^ partition into the organic or aqueous phase while error bars indicate the CI (N = 3). The left 2 bar graphs reveal that 500 nM C2d does not chelate Fe^2+^ as extraction by hexane leaves the ion only in the aqueous phase. The right 2 bar graphs indicate that 500 nM hinokitiol does chelate Fe^2+^ as extraction by hexane leads to 34.7 ± 0.5% of the Fe^2+^ present in the hexane layer. **b** Fe^3+^. The bars’ lengths indicate how much of the original 200 nEq/L of ^59^Fe^2+^ partition into the organic or aqueous phase while error bars indicate the 95% CI (N = 3). The left 2 bar graphs reveal that 500 nM C2d does not chelate Fe^3+^ as extraction by hexane leaves the ion only in the aqueous phase. The right 2 bar graphs indicate that 500 nM hinokitiol does chelate Fe^3+^ as extraction by hexane leads to 11.0 ± 0.5% of the Fe^3+^ present in the hexane layer. **c** Mn^2+^. The bars’ lengths indicate how much of the original 350 nEq/L of ^54^Mn^2+^ partition into the organic or aqueous phase while error bars indicate the 95% CI (N = 3). Both 500 nM C2d and 500 nM hinokitiol yield similar results with all Mn recovered in the aqueous phase

It is also important to learn how hinokitiol interacts with other metal ions, given their physiological and toxicological implications. Rather than develop assays for radioactively tagged metal ions, we adapted another assay. Grillo et al. (2017) also showed that hinokitiol is able to partition metal ions into a lipid layer after chelating them. We used this assay to verify our results with Fe and Mn ions paralleled the data on import of these metal ions, then extended it to using other metal ions. Figure 5 illustrates results for ^59^Fe^2+^, ^59^Fe^3+^ and ^54^Mn^2+^ analyses.

These results with hinokitiol also confirm and extend those obtained by Grillo et al. (2017) showing that ferrous (Fig. 5a) and ferric (Fig. 5b) iron both bind to hinokitiol rendering the iron able to partition to the organic phase. Consistent with more effective import by hinokitiol of ferrous (Fig. 1c) than ferric (Fig. 2c but note the y-scale) iron, ferric iron extracts less well after binding to hinokitiol. Based on the transport results, one might also expect that hinokitiol does not chelate Mn^2+^ without an excess of the chelator so that it will not appear in the organic phase. This expectation was tested and met (Fig. 5c).

Because of the regulatory complexity of working with radioisotopes for the other metals of interest, we chose to modify this extraction assay to have the results ascertained via inductively coupled plasma-optical emission spectrometry (ICP-OES). First, however, we examined the concentration dependence in the assay above to assure that the higher concentrations needed for ICP-OES should produce interpretable results. Supplementary Fig. S2 portrays these analyses with the data indicating that C2d binds neither ferrous (S2a) nor ferric (S2b) ions consistent with Figs. 1b and 2b, respectively, where the control does not support their import. Fig. S2 shows that hinokitiol, however, does bind the two ions with ferrous (S2a) clearly associating more effectively than ferric (S2b), consistent with the Km results in Table 1.

The next experiments tested whether the results for ICP-OES were equivalent to those with radioisotope tags for the three metal ions already examined. Supplementary Fig. S3 illustrates results for Fe^2+^ analysis. The bar graph like Fig. 5a supports the distinction between hinokitiol as chelating ferrous ions and allowing their extraction into an organic phase versus the negative control that does not chelate them is a property that reproduces over at least a 5000-fold range of Fe^2+^ concentrations. We surveyed some of the metals ultimately tested at this level (10 µEq/L with 20 µM hinokitiol) and were usually unable to detect partitioning (data not shown). Because the Burke group found that they had to use higher, non-physiological concentrations of most other metals, we also tested similar high levels; data for Cd^2+^ are representative (Supplementary Fig. S4). The results show that statistically significant amounts of this toxic ion appear in the organic phase when hinokitiol is present; but the actual quantity is close to minimal. These data are summarized in Table 2 as also are similar data for Pb^2+^; they indicate that accumulation of toxic levels of either metal ion due to hinokitiol substituting for DMT1 is not a likely problem.

**Table 2:** Extraction of metal ions into hexane

Metal ion	Hinokitiol	C2d	*P*	*N*
200 nM ^59^Fe^2+^	34.7 ± 0.5	0 ± 0.1	< 0.0005	3
200 nM ^59^Fe^3+^	11.0 ± 0.5	0 ± 0.3	< 0.0005	3
350 nM ^54^Mn^2+^	0 ± 0.60	0 ± 0.58	0.985	3
10 μM Fe^2+^	145 ± 89	− 5.5 ± 4.6	0.0006	6
100 μM Cd^2+^	0.04 ± 0.02	− 0.001 ± 0	0.0028	3
100 μM Pb^2+^	0.181 ± 0.14	0.01 ± 0.1240	0.0039	6
100 μM Mn^2+^	0.014 ± 0.01	0 ± 0.002	0.0032	6
100 μM Cu^1+^	0.02 ± 0.012	0.005 ± 0.006	0.0038	6
100 μM VO^2+^	0.003 ± 0.002	− 0.001 ± 0.006	0.086	6
100 μM Zn^2+^	0.016 ± 0.026	− 0.008 ± 0.008	0.009	6
100 μM Cu^2+^	0.002 ± 0.01	− 0.009 ± 0.002	0.009	6
100 μM Co^2+^	0.031 ± 0.016	− 0.003 ± 0.004	0.17	6
100 μM Ni^2+^	− 0.013 ± 0.004	− 0.012 ± 0.002	0.51	6

The top 3 rows of data come from Fig. 5; the next 2 rows, from Supplementary Figs. S3, S4. Remaining rows came from similar analyses (not shown)

These results with hinokitiol also confirm and extend those obtained by Grillo et al. (2017). The portion of ^59^Fe^2+^ that is extracted with C2d is 0; while with hinokitiol it is 34.7 ± 0.5% (Table 2). These results illustrate the properties of hinokitiol that permit it to substitute for DMT1 in ferrous ion import. One might also expect that hinokitiol must chelate Fe^3+^ so that it too will appear in the organic phase. This expectation was tested in a similar fashion; again the control does not lead to any counts in the organic phase while hinokitiol retrieves 11.0 ± 0.5% (Table 2). Mn is clearly not transported nor is it extracted (Table 2) until non-physiological levels are present. Whereas DMT1 imports multiple other metal ions of physiological or toxicological relevance and has also been tested for other ions that remain controversial, Table 2 indicates that substituting hinokitiol for DMT1 is unlikely to cause import any of these ions to an extent that will ordinarily impact their metabolic behavior.

## Discussion

### Can hinokitiol substitute for DMT1 and does that ability depend on a ferrous ion concentration gradient?

The initial premise for this project was that an ion gradient generated to reveal DMT1 activity would also reveal the ability of hinokitiol to import that ion, here Fe^2+^. Initial support for that premise came from how well the small molecule substituted for the major iron importer when conditions were chosen to assess DMT1 activity (Fig. 1). Given that hinokitiol alleviates the anemia of the Belgrade rat (Grillo et al. 2017) and that the anemia derives from a G185R mutation in DMT1 (Fleming et al. 1998), this result is not surprising but its novelty depends on the gradient here coming from experimental intervention rather than the mutational loss of the gene product. The next experiments compared some of the ions with which the transporter and the small molecule interacted.

### Metal ion interactions, round 1

While hinokitiol binds to and supports the import of ferric ions, DMT1 does not (Fig. 2). This result is a reminder that nearly two decades have passed since clear published results showed that there were separate pathways for ferrous and ferric iron import (Conrad et al. 2000), yet only the path for the former has seen more publications to elaborate on it. The participants in the latter pathway remain largely uncharacterized except to the extent that multiple ferrireductases that divert Fe^3+^ into the Fe^2+^ pathway are now known (McKie et al. 2001; Ohgami et al. 2006; Tripathi et al. 2015). Hinokitiol, however, can import Fe^3+^ without prior reduction.

The situation is also different for Mn^2+^ (Fig. 3) with DMT1 effectively importing this metal ion, but hinokitol unable to do so until the level of Mn^2+^ (Table 2) is far above what is physiological. The potential for DMT1 to transport Mn in addition to iron was initially noted in the study that led to the acronym DCT1 (Gunshin et al. 1997)—for divalent cation-transporter 1—but it soon generated a better appreciation for how iron and manganese metabolism interact in both physiological and toxic fashions (Roth and Garrick 2003). One would therefore not expect hinokitiol to have a direct impact on these interactions in the ways that DMT1 does.

### Transport kinetics

The original treatment of Eq. 2 (Michaelis and Menten 1913) relied on the assumption that [S] ≫ [E] so that one could use the input concentration of S as [S]. If, however, one considers that the activities of S and E are the more chemically appropriate values, this treatment might still apply [as well as the linearization in Eq. 3 (Lineweaver and Burk 1934)] despite our using concentrations of hinokitiol not much below the range (~ 3–4 μM) of the Km for DMT1. We chose therefore to find out whether similar saturation curves and linearizations were the result for varying [Fe^2+^], asking if they were similar to those for DMT1 (Garrick et al. 2006a). They were (Fig. 4, Supplementary Fig. S1) and the Km’s (Table 1) even indicated that hinokitiol had a higher affinity for Fe^2+^ by nearly an order of magnitude. We also then did similar analyses for Fe^3+^ to find that the affinity of hinokitiol for these ions was × ~ 5 less (Table 2). Because it was easy to vary the level of the hinokitiol, we also did Km studies for it. We again observed a saturable process (Table 2). It is beyond the scope of the current paper to address whether the process was due to limiting constant quantities of the substrate or another component of the system like the ability to solubilize hinokitiol in the plasma membrane. It is novel to find that this type of kinetic analysis can be applied to a small molecule substituting for the transporter and hence inverted between the catalyst and the reactant.

### Metal ion interactions, round 2

There have been many studies showing that DMT1 is relatively promiscuous in terms of which metal ions it can transport (Arredondo et al. 2003, 2014; Bannon et al. 2002, 2003; Davidson et al. 2005; Garrick et al. 2003, 2006a, b; Gunshin et al. 1997; Illing et al. 2012; Jiang et al. 2013; Knöpfel et al. 2005; Roth and Garrick 2003) while dysfunction after mutation implies it serves iron homeostasis primarily (Fleming et al. 1997, 1998; Gunshin et al. 2005). The consensus adds Cd^2+^, Mn^2+^, Co^2+^, Ni^2+^, Pb^2+^, VO^2+^ and Zn^2+^ to the metal ions it can transport, while excluding Fe^3+^ and Cu^2+^. Cu^1+^ has seen some debate but current support looks sufficient (Arredondo et al. 2003, 2014; Jiang et al. 2013). Clearly this promiscuity indicates that DMT1 not only serves iron import but also involves some toxic risks and may support other metal ion transporters by providing some redundancy to exploit. We therefore asked if hinokitiol was similarly promiscuous. To screen the variety of cations, we verified that the combination of lipophilicity and chelation as properties of hinokitiol (Grillo et al. 2017) to which the Burke lab attributed its ability to substitute for DMT1 did parallel our positive metal ion import results with radioisotope tagged Fe^2+^ and Fe^3+^ (Fig. 5, Supplementary Fig. S2) and the negative results with Mn^2+^ (Fig. 5). Adopting a variation of the assay relying on ICP-OES, we still saw consistency with the Fe data (Supplementary Fig. S3) and established that hinokitiol works, but minimally for the toxic metal ion, Cd^2+^ (Supplementary Fig. S4). To the extent that one can rely on this partition assay, our data do not support physiological transport of the other metal ions tested in Table 2. Thus we conclude that hinokitiol has a narrower substrate specificity than DMT1, a property that may be an advantage in terms of interacting with the homeostasis of these metal ions and particularly in terms of not importing toxic metals like Cd and Pb. Hinokitiol’s ability to import Fe^3+^ could pose an issue relative to iron overload but clearly Fe^2+^ import by hinokitiol is more effective than for Fe^3+^ so a little caution in monitoring iron status while using hinokitiol appears to be what this property calls for rather than excluding the use of hinokitiol.

## Conclusions

Hinokitiol relies as predicted on concentration gradients of Fe^2+^ and Fe^3+^ similar to those needed to assay for DMT1 or those generated when DMT1 deficiency exists. Because hinokitiol is serving in the place of a transporter, the same type of kinetic analyses that apply to transporter kinetics also serve to describe metal ion import dependent on hinokitiol. Hinokitiol is less promiscuous in interacting with metal ions than DMT1; this property should help in determining whether the smaller molecule can be used as a gene therapy replacement for genes involved in iron homeostasis like *SLC11A2.*

## Materials and methods

### Materials

^59^FeCl_3_ (NEZ0375) and ^54^MnCl_2_ (NEZ040) were purchased from Perkin Elmer (Boston, MA) supplied in 0.5 M HCl. Hinokitiol and C2doxyhinokitiol were synthesized in the Burke laboratory as described (Grillo et al. 2017). HEK293 cells permanently transfected with 1A/+ IRE or 1B/− IRE DMT1 subject to tet-on regulation have been described (Garrick et al. 2006a).

### Incubations

Briefly, HEK293 cells were grown to 70–80% confluence in 6 well plates then treated with doxycycline (when DMT1 activity was the goal) or just its solvent (when endogenous transport was the goal) then grown fully to confluence (24–48 h) all as described before (Garrick et al. 2006a). Incubations also included 500 nM hinokitiol or C2d for the periods indicated and ^59^Fe^2+^, ^59^Fe^3+^ or ^54^Mn^2+^ with the labeling conditions also all as described before (Garrick et al. 2006a). When determining the kinetics, we varied either the iron concentration or the hinokitiol concentration while holding the hinokitiol or the iron concentration constant, respectively, as indicated in the figure or table legends.

### Extractions

For ^59^Fe experiments, [Fe] = 200 nEq/L in Hank’s Buffered Saline Solution (HBSS) unless otherwise specified; while hinokitiol and C2d were 500 nM. As iron binding of hinokitiol is tridentate, the concentrations chosen provided a modest excess of Fe. When Fe^2+^ was present, this status was achieved by having a  × 10 excess of FeSO_4_ relative to the ^59^Fe and a  × 10 excess of ascorbic acid relative to the FeSO_4_. When Fe^3+^was present, this status was achieved by having a  × 10 excess of ferric ammonium citrate (FAC) present relative to the ^59^Fe. For ^54^Mn experiments, [Mn] = 350 nEq/L was achieved by adding MnCl_2_. All extractions involved 1 mL for the aqueous phase and an equal volume of hexane. Tubes were vortexed at low speed, allowed to settle and aliquots of the two phases removed and allowed to dry in a fume hood then counted in a LKB Compugamma 1282 γ counter.

For nonradioactive experiments, the metals were at 10 or 100 μEq/L in HBSS; while hinokitiol and C2d were 20 or 200 μM, respectively, leading to a slightly larger excess of metal ion than in the radioactive extractions. Metals were provided as FeSO_4_ (with a  × 10 excess of ascorbate), FAC, MnCl_2_, Pb(CH_3_CO_2_)_2_, Cd(CH_3_CO_2_)_2_, CoCl_2_, NiCl_2_, VOSO_4_, Cu(CH_3_CO_2_)_2_ with a  × 10 excess of histidine, Zn(CH_3_CO_2_)_2_ and CuSO_4_ with both histidine and ascorbate in  × 10 excess. Extractions were performed as above but metals were determined using ICP-OES (Model Optima 4300D, Perkin Elmer, Norwalk, CT) after drying the two phases in a fume hood and dissolving an aliquot of each in 3 N HCl/10% trichloracetic acid.

### Statistical analysis

Statistical analysis were done with Stata version 15.1 (StataCorp, College Station, TX). Although *P* < 0.05 was treated as significant, we report *P* where possible. We used no correction for multiple statistical tests when making them because we tested only experimental hypotheses but readers should be aware that this approach was employed.

## Electronic supplementary material

Below is the link to the electronic supplementary material.

Supplementary material 1 (DOCX 42 kb)
