# Supplementary material for: A direct comparison of divalent metal-ion transporter (DMT1) and hinokitiol, a potential small molecule replacement
Source: Biometals. 2019 Jul 31;32(5):745–55. doi: 10.1007/s10534-019-00207-2 (PMC6768898; doi:10.1007/s10534-019-00207-2)
Supplement: Supplementary file 1 — Supplementary material 1 (DOCX 42 kb) [file 10534_2019_207_MOESM1_ESM.docx]

**Supplementary Materials**

Fig. S1


Caption Fig. S1. Lineweaver-Burk plot for the reciprocals of the axes of Fig.4. The estimates for Km = 242 nEq/L and Vmax = 3.5 nEq/(Lxmin).

Fig. S2


Caption Fig. S2. Concentration dependence for iron with hinokitiol / hexane extraction. a. Fe^2+^ *versus* b. Fe^3+^. Open circles indicate for 500 nM hinokitiol how much of the original nEq/L of Fe partitions into the organic or aqueous phase while error bars indicate the CI (N=3) while open triangles confirm that 500 nM C2d does not chelate either ionic Fe as extraction by hexane leaves the ion only in the aqueous phase. Note that both axes have been rescaled.

Fig. S3


Caption Fig. S3. Box plots testing whether hinokitiol allows Fe^2+^ extraction. Medians and interquartile range are indicated with whiskers showing adjacent values; solid circles indicate points within this range while open circles indicate outliers. The medians indicate how much of the original 10 μEq/L of Fe^2+^ (presented as FeSO_4_ in the presence of a 10x excess of ascorbate) partitions into the organic or aqueous phase (N=6). The left 2 plots confirm that 20 μM C2d does not chelate Fe^2+^ as extraction by hexane leaves the ion only in the aqueous phase. The right 2 plots indicate that 20 μM hinokitiol does chelate Fe^2+^ as extraction by hexane leads to 145 ± 89 ppm of the Fe^2+^ present in the hexane layer for hinokitiol *vs.* -5.5 ± 4.6 for C2d (*P* = 0.00062 by Wilcoxon rank-sum test).

Fig. S4


Caption Fig. S4. Box plots testing whether hinokitiol allows Cd^2+^ extraction. Medians and interquartile range are indicated with whiskers showing adjacent values; solid circles indicate points within this range while open circles indicate outliers. The medians indicate how much of the original 100 μEq/L of Cd^2+^ (presented as Cd(Acetate)_2_) partitions into the organic or aqueous phase (N=6). The left 2 plots confirm that 200 μM C2d does not chelate Cd^2+^ as extraction by hexane leaves the ion only in the aqueous phase. The right 2 plots indicate that 200 μM hinokitiol chelates Cd^2+^, but marginally as extraction by hexane leads to 0.04 ± 0.02 ppm of the Cd^2+^ present in the hexane layer for hinokitiol *vs.* -0.001 ± 0 for C2d (*P* = 0.0028 by Wilcoxon rank-sum test).
